# Presence of continental slivers in oceanic transform faults determined by rift inheritance

**DOI:** 10.1038/s41561-025-01795-0

**Published:** 2025-09-25

**Authors:** Attila Balázs, Taras Gerya, Gábor Tari

**Affiliations:** 1 Department of Earth and Planetary Sciences, Institute of Geophysics, Geophysical Fluid Dynamics Group, ETH Zürich, Zürich, Switzerland; 2 OMV Energy, Vienna, Austria

**Keywords:** Tectonics, Geodynamics, Structural geology

## Abstract

The theory of plate tectonics describes how continents are separated from each other by lateral movement that is accommodated by transform faults connecting mid-ocean ridge sections, which leaves inactive fracture zones on the ocean floor. The occurrence of continental crustal slivers in these fracture zones at distances of hundreds of kilometres to 1,000 kilometres from continents has been documented worldwide, yet their occurrence is not expected from classical plate tectonic theory. Here we use three-dimensional magmatic-thermomechanical numerical simulations to investigate the transition from continental rifting to the birth of oceanic transform fault zones and their relationship to mantle melting and crustal tectonics. These simulations show that continental slivers are entrapped within shear zones in the oceans inherited from preceding continental rifting stage. They also show three distinct stages of transform fault zone formation—continental rift linkage, proto-transform, oceanic transform—resulting from progressive strain localization into a narrowing extension-parallel strike-slip shear zone. Additionally, continental sliver emplacement into oceanic lithosphere is shown to be associated with specific stages of subsidence and uplift linked to the changing transtensional and transpressional stress field, thereby notably modifying the ocean floor morphology, mid-ocean ridge melting conditions and transform fault seismicity.

## Abstract (web summary)

Slivers of continental crust within oceanic transform faults far from continents result from their entrapment within shear zones during the early stages of continental rifting, according to three-dimensional magmatic-thermomechanical numerical simulations.

## Main

The bimodal distribution of Earth’s global topography reflects the presence of both continental and oceanic crust. Continental rifting is a fundamental process that separates continental plates through progressive crustal and lithospheric thinning, ultimately leading to mid-ocean ridge formation, where new oceanic crust is generated^1,2^. Mid-ocean ridges are segmented and connected by transform faults, traditionally described as two-dimensional strike-slip boundaries accommodating relative horizontal motions between tectonic plates of different thermal ages^3^. Global-scale modelling showed that plate tectonics and the toroidal component of surface motion necessarily leads to the development of diffuse or localized transform plate boundaries^4^. Analogue modelling of freezing wax experiments reproduced localized, orthogonal ridge-transform patterns of oceanic plate separation^5^.

Recent observations and modelling^6^ inferred a dynamic evolutionary model of ridge-transform segments driven by dynamical instabilities^7^, maintaining oblique extension in the transform valleys linked to deepening of their seafloor^8^. In addition, detailed seafloor mapping along oceanic transform and fracture zones also revealed bathymetric highs aligned parallel with the principal transform zones. Their formation is commonly linked to extension and flexural vertical adjustments forming transverse ridges^9^ or linked to plate velocity-reorganization-related convergence and uplift^10,11^. Furthermore, slivers of the continental crust were reported or inferred within oceanic transform faults worldwide^12–15^, but the physical mechanisms that drive the emplacement of such continental pieces in the oceans remained controversial. Previous models of continental rifting have addressed the formation of microcontinents—larger, isolated tectonic fragments of continental crust surrounded by oceanic crust—and continental ribbons that are slightly extended continental blocks still attached to unrifted continental margins^16,17^. Such models discussed the role of mantle plumes, ridge jumps and rift jumps during various stages of stretching, thinning and exhumation. However, they do not resolve the link between oceanic transform fault zone formation, pre-existing continental inheritance and the emplacement of narrow, elongated continental slivers into the oceanic domain, bounded by fossil or active transform faults, often located far from rifted margins. Here we show, using numerical modelling and observations from incipient and mature oceanic transform fault zones, how their evolution is connected to their preceding continental rifting and break-up stages. We propose an evolutionary model that explains the successive stages of oceanic transform fault zone localization, including stages of transtensional and transpressional deformation in a gradually narrowing shear zone, where pre-rift continental slivers are entrapped, preserved and transported.

## Ocean floor morphology and intra-oceanic continental slivers

**Fig. 1:**
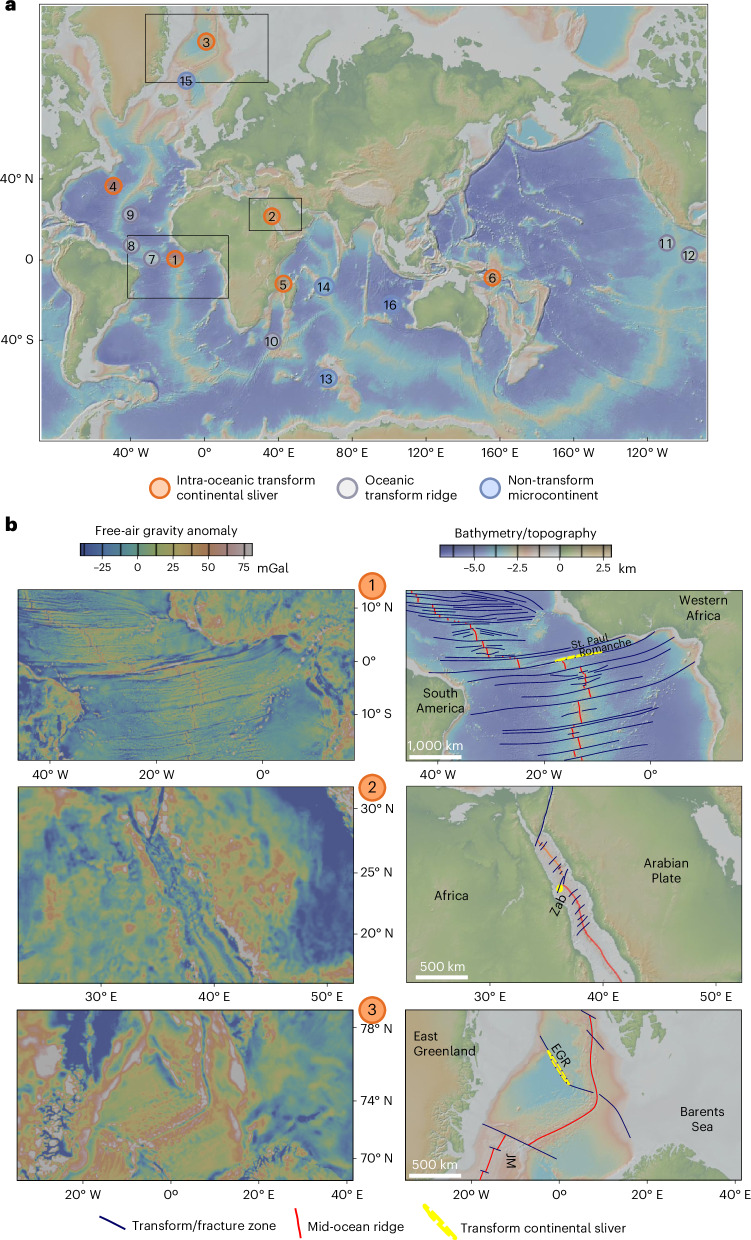
Selected transform continental sliver, oceanic transform ridge and microcontinent occurrences shown by orange, grey and blue circles, respectively, in various oceanic locations. **a**, Global bathymetric and topographic map showing locations of the three genetically distinct types of ocean floor bathymetric feature. (1) Continental crustal slivers in the Romanche oceanic transform fault zone, (2) Zabargad continental island (Zab) in the Red Sea basin, (3) East Greenland Ridge (EGR), (4) Newfoundland Ridge in the northern Atlantic Ocean, (5) Davie continental sliver, (6) Woodlark Basin sliver. Selected transverse and median transform ridges: (7) St. Peter and St. Paul islands, (8) Vema Transverse Ridge, (9) Kane Transform in the Atlantic, (10) Mitin Ridge in the Indian Ocean, (11) Clipperton Transform Ridge, (12) Siqueiros Transform. Microcontinents: (13) Elan Bank of the Kerguelen Plateau, (14) Seychelles, (15) Jan Mayen and (16) Gulden Draak Knoll microcontinents. **b**, Free-air gravity anomaly and bathymetric maps of three continental sliver occurrences in different oceanic transform evolutionary stages marked by yellow colour.

**Extended Data Fig. 1:**
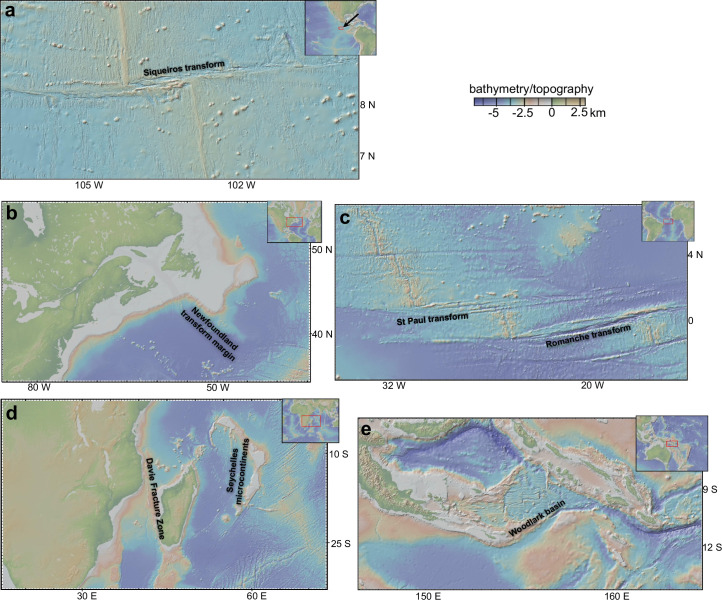
Bathymetry of selected transform fault zones. **a**, intra-transform spreading in the Siqueiros transform. **b**, Transform margin of Newfoundland. Note the shallow bathymetry of the continental domain extending into the deep ocean. **c**, Romanche and St Paul transform fault zones. Note the sigmoid shape of the transpressive St Paul Island and the elongated ridges in the Romanche transform. **d**, Transform ridges within the Davie Fracture zone. **e**, Woodlark basin. Note the shallow bathymetric features along the transform faults. Basemaps compiled by GeoMapApp^49^.

Oceanic active transform fault zones and their associated inactive fracture zones display a wide range of bathymetric features (Fig. 1 and Extended Data Fig. 1). Some transform valleys reach depths exceeding 7,000 metres (ref. ^6^) and are flanked by steep walls rising several kilometres. The bathymetry of such oceanic transform fault zones is controlled by divergence rate and magma supply but is also influenced by variations in transform fault orientation and the stress field, which create localized transtensional or transpressional regimes^18–21^. In some cases, extensional deformation within transform faults leads to the formation of new spreading centres, resulting in oblique spreading—such as in the Siqueiros transform-fault system^10,22,23^.

Bathymetric ridges along oceanic transform faults occur in two main settings: as transverse ridges bordering the fracture zone valley, often with a steep flank facing the fracture zone or as median ridges within the transform valley itself (Fig. 1a). Transpressional uplift of such zones was proposed to be controlled by plate velocity and spreading re-organization, such as in the Romanche or St. Paul transforms in the equatorial Atlantic^24^. For instance, ridge overlap at the St. Paul transform led to the uplift of the St. Peter and St. Paul islets approximately 10 million years ago, exposing mantle rocks^11^.

The Romanche megatransform, characterized by a 900-km offset and a 100-km-wide transform valley, has been active since the separation of Africa and South America, as evidenced by its fracture zone extending between the conjugate rifted margins (Fig. 1). Transverse ridges along this system, located at depths as shallow as 875–1,000 metres, are capped by ~300-metre-thick carbonate platforms^25^, suggesting episodes of uplift, exposure and subsequent thermal subsidence^26^. Additionally, dredging has recovered Late Tithonian–Early Berriasian (~147–144 Ma) sediments and metasediments from these ridges^13^. These continent-derived sediments are juxtaposed against oceanic crust aged 60 Ma to the north and <25 Ma to the south. Their presence within the transform system led to the concept of continental slivers embedded within oceanic transform faults^12,13,27^.

We identify and interpret similar continental crustal slivers within both juvenile and mature oceanic transform fault zones globally (Fig. 1 and Extended Data Table 1). The Zabargad Island in the Red Sea lies within the Zabargad Fracture Zone, a broader shear zone that offsets active spreading ridges. The fracture zone extends between rifted margins, indicating activity since crustal break-up^28^. Exposed rocks on Zabargad Island include Pan-African granitic gneisses, basaltic dykes, gabbro intrusions and sedimentary units containing pre-rift Cretaceous deposits^29^. The island’s uplift is interpreted as the result of transpression along the Zabargad Fracture Zone^14^. Another example is the East Greenland Ridge in the Northern Atlantic (Fig. 1). A 200-km long, 20–30-km wide continental sliver is studied by detailed seismic and gravity methods^15^. Its formation is connected to the oblique continental rifting and transform evolution along the Senja and Greenland Fracture Zones^15^^,30^. This sliver is probably still attached to the East Greenland rifted margin. Similarly, the Newfoundland Ridge along the Southern Newfoundland Transform Margin represents a wider continental spur linked to transform margin formation during the breakup of Iberia, the Grand Banks and Northwest Africa^31^. Narrow continental slivers are located within the Davie Fracture Zone^32,33^, being confirmed by dredging and inferred in the Woodlark Basin^34^ based on detailed geophysical mapping (Fig. 1). In all these cases, continental sliver emplacement is closely tied to the formation and evolution of oceanic transform fault zones.

## Continental sliver emplacement into oceanic transform faults

**Fig. 2:**
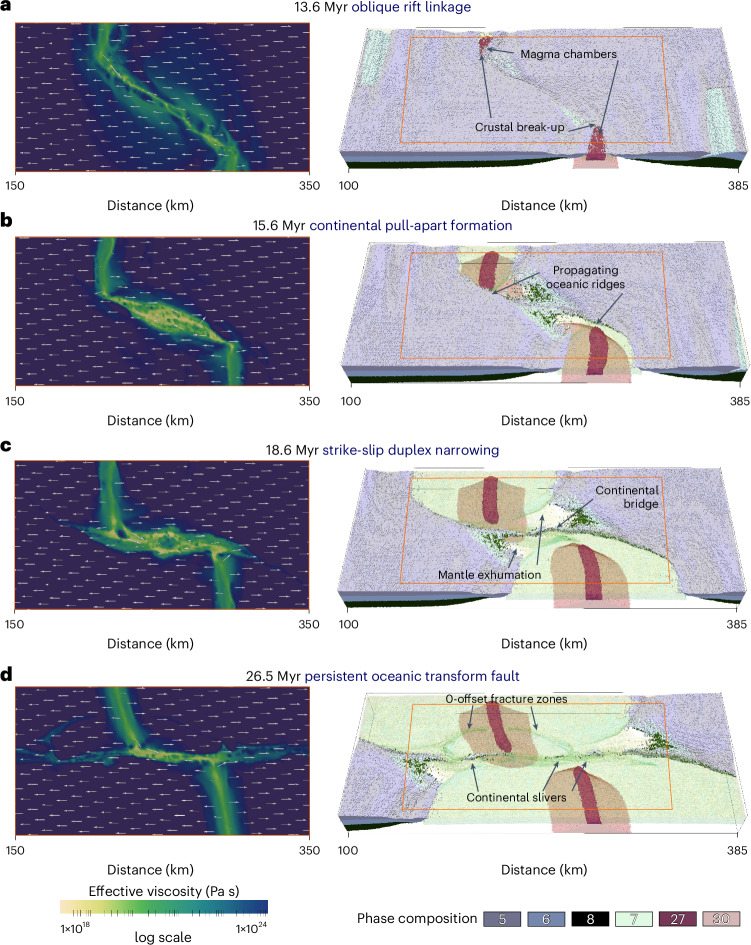
Numerical evolutionary model of the formation and evolution of continental slivers in oceanic transform faults. Results are simulated by 3D magmatic-thermomechanical geodynamic and coupled surface processes models and shown by active deformation and velocity field on horizontal maps and rock composition (set-up is shown in Extended Data Fig. 2; strain rate maps are shown in Supplementary Figs. 1 and 2). **a**, Oblique rifting. **b**, Formation of a crustal-scale pull-apart basin. **c**, Strike-slip duplex formation. **d**, Oceanic transform fault formation. Sediments are transparent in this figure. Phase composition: (5) continental upper crust, (6) continental middle crust, (7) oceanic upper crust (basalt), (8) continental lower crust, (27) molten basalt, (30) partially molten peridotite.

**Fig. 3:**
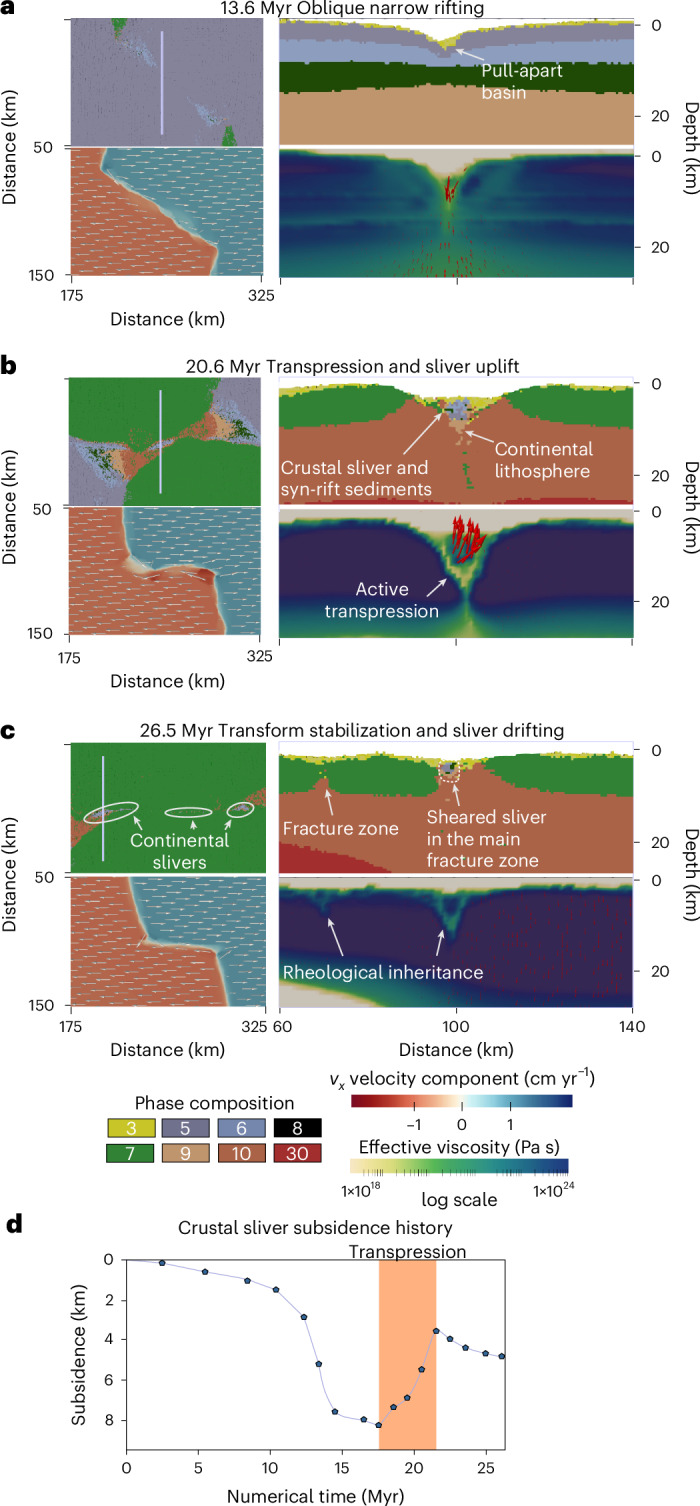
Continental crustal sliver formation, preservation and velocity field. **a**–**c**, Numerical model evolution shown by top-view phase composition and velocity maps of the *x* horizontal velocity component (*v*_*x*_; left) and by vertical cross sections of the phase composition and effective viscosity (right). **a**, Oblique rifting stage. **b**, Short-lived transpression within the transform zone. **c**, Persistent transform faulting. Phase composition: (3) sediments, (5) continental upper crust, (6) continental middle crust, (7) oceanic upper crust (basalt), (8) continental lower crust, (9) lithospheric mantle, (10) asthenospheric mantle, (30) partially molten peridotite. **d**, Subsidence history of the crustal sliver in the transform zone.

**Extended Data Fig. 2:**
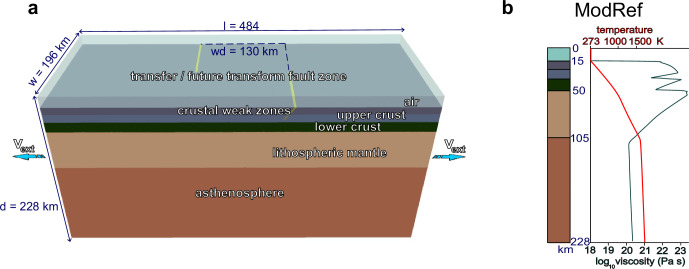
Initial numerical model setup. **a**, Initial 3D rock compositional layering. **b**, Initial effective viscosity and temperature profile of the reference model.

We conducted a series of three-dimensional (3D) magmatic-thermomechanical geodynamic modelling to simulate continental rifting and subsequent seafloor spreading, incorporating surface evolution, erosion and sedimentation, nonlinear viscoplastic rheologies and mantle partial melting processes (Methods and Extended Data Fig. 2). Our goal was to investigate the emplacement of continental slivers into oceanic transform fault zones (Figs. 2 and 3).

The models show three distinct stages of oceanic transform fault formation—continental rift linkage, proto-transform and eventually oceanic transform stages. The process begins with rift initiation along two inherited weak zones placed in an en-echelon geometry in the crust along the frontal and rear model boundaries. This configuration resembles geological reconstructions proposed for the Central Atlantic or the East Africa and Madagascar rifting history^33,35^. In the transfer zone between the weak zones in the centre of the model, initial deformation is more distributed. In the reference model simulating a total rate of 1.9 cm yr^−1^ plate divergence, crustal break-up is reached after 13 Myr of lateral rift migration along the frontal and rear model sides (Figs. 2a and 3a). This leads to the formation of two mid-ocean ridges connected by a continental oblique shear zone in the transfer zone. Rapid subsidence and sedimentation take place in narrow pull-apart basins (Fig. 3a). The strike of the main shear zone connecting the mid-ocean ridges gradually rotates into an extension parallel orientation as crustal break-up and the mid-ocean ridge segments propagate closer in the transfer zone. Oceanic crust is continuously formed along the active mid-ocean ridges. By 15.6 Myr the transfer zone evolves into one crustal-scale pull-apart basin floored by thinned continental lithosphere (Fig. 2b). The deformation zone connecting the oceanic domains narrows gradually and by 18.6 Myr, an ~20-km wide strike-slip duplex bounds the last continental bridge connecting the drifting continental margins (Fig. 2c). Distributed deformation in the transfer zone governs locally lower mantle thermal gradients that delays mantle melting leading to mantle exhumation after crustal break-up (Figs. 2c and 3b). The two mid-ocean ridge segments then propagate closer in the centre, forming a transient overlap geometry that induces a short-lived transpressional stage in the transfer zone, rapidly uplifting the continental crustal sliver (Fig. 3b,d). Eventually, the transfer deformation zone evolves into a narrow extension parallel strike-slip zone, following the short events of transtensional and locally transpressional stages (Fig. 3). The sheared remnants of the continental bridge are distributed in the active transform fault zone connecting the mid-ocean ridge segments, and eventually they are transported into the inactive fracture zones (Figs. 2d and 3c). These continental slivers maintain a minimum thickness of ~5–7 km, underlain by a thin remnant of continental lithospheric mantle.

## Drivers of continental sliver formation and preservation

The occurrence of continental slivers in oceanic transform faults is inherently linked to the rifting history. The rejuvenation of inherited lithospheric-scale structures, that is, suture zones or crustal-scale thrust or nappe contacts during the subsequent stages of continental rifting has been in the forefront of research since the birth of the Wilson-cycle concept^2^. Geological studies, geophysical imaging and geodynamic modelling showed that the potential for selective reactivation of shear zones depends on the orientation of fossil crustal and mantle structures and fabrics, variable plate kinematics and deep mantle upwellings^20,36–39^. The diachronous reactivation of inherited structures and the migration of deformation—leading to the development of en-echelon rift structures and perpendicular, continental proto-transform zones that eventually control the formation of intra-oceanic continental slivers—have been proposed in several regions, including the equatorial Atlantic^35^ or the East Africa–Madagascar rifted and transform margins^32^. Furthermore, a single rift propagator can also entrap pieces of continental crust as in the Red Sea^14^ or Woodlark Basin margins^34^. Rift propagation, obliquity and segmentation are linked to interaction with inherited structures and modulated by compressional and transpressional stress fields^40,41^. Therefore, occurrences of intra-oceanic continental slivers are expected in transform and fracture zones, whose proto-transform evolution was coupled to their continental rifting stage, as in many cases in the Atlantic^35^, whereas no slivers are expected in the Pacific, where the formation of most oceanic transform fault zones post-date crustal break-up.

During continental rifting, the efficiency of rift linkage is controlled by lithospheric rheology and the lateral distance between the propagating rift zones governing stress transfer and strain localization^41–43^. When the across-strike distance between the rift zones is small, a continuous oblique-slip zone develops. In contrast, larger distances and less efficient strain localization in the transfer zone prevents transform fault formation and leads to ridge jumps, which favour microcontinent formation^44^. Examples include the Jan Mayen microcontinent in the North Atlantic, the Kerguelen Plateau or the Seychelles microcontinents in the Indian Ocean, where plume activity supported their formation^45,46^ (Fig. 1). When the lateral distance between the propagating rift zones is in the order of the lithospheric thickness, a stable oceanic transform fault zone develops eventually^20,44^. In this case, the later strain localization in the transfer zone and the gradual formation of a strike-slip shear zone bounds the narrow, continuous continental bridge connecting the drifting continental plates. Following final break-up in the transfer zone, thinned segments of the previous continental bridge remain attached to the rifted margins, showing similar geometries as imaged along the East Greenland Ridge or the Newfoundland Ridge, whereas sheared continental slivers are also present along the oceanic transform and fracture zones. The eventual formation of a persistent, extension-parallel transform fault zone, containing remnants of continental crustal slivers, required the high-resolution implementation of rheological weakening mechanisms, such as plastic strain weakening or mantle grain-size evolution^47^.

**Fig. 4:**
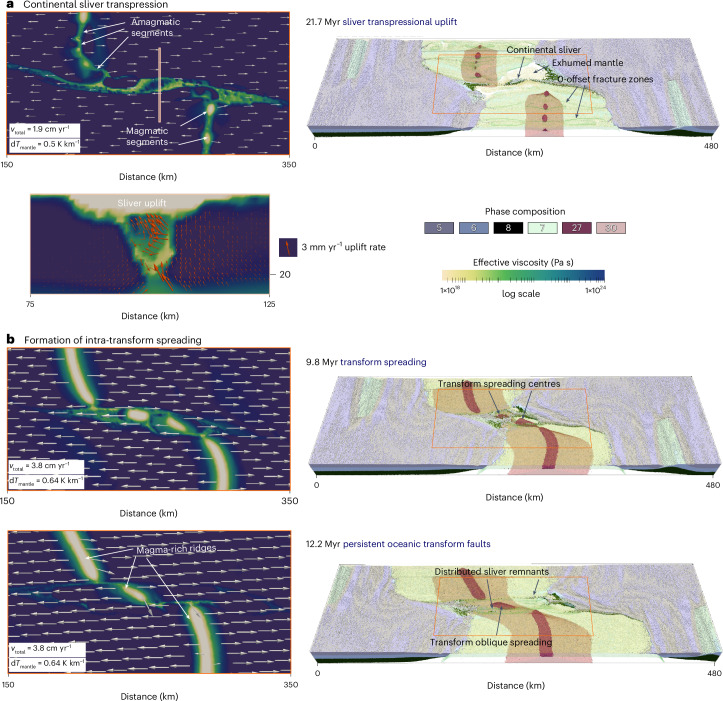
Contrasting transform evolution controlled by different thermal gradients resulting in intra-transform spreading or transform sliver formation. Results are simulated by 3D magmatic-thermomechanical geodynamic and coupled surface processes models and shown by highlighting the active deformation and velocity field on horizontal maps and a vertical cross-section (left) and rock composition (right). **a**,Model simulation with a lower thermal gradient. **b**,Model simulation with faster plate divergence and a higher thermal gradient. Phase composition: (5) continental upper crust, (6) continental middle crust, (7) oceanic upper crust (basalt), (8) continental lower crust, (27) molten basalt, (30) partially molten peridotite. *v*_total_is the total divergence velocity; d*T*_mantle_is the mantle thermal gradient.

**Fig. 5:**
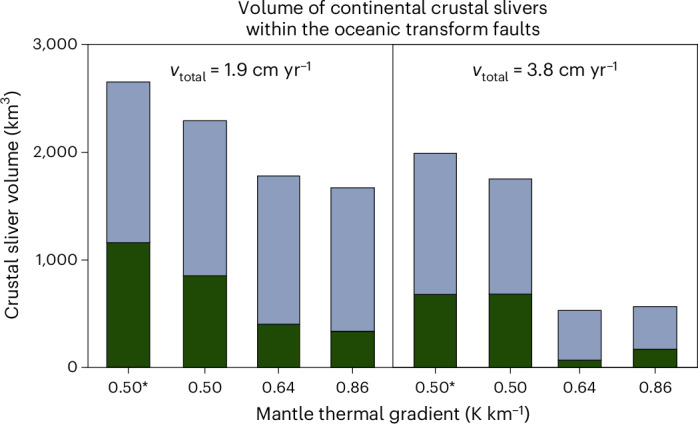
Volume of crustal slivers within the modelled transform fault zones in different numerical models. Light grey colour indicates the continental upper and middle crust; dark green colour indicates the continental lower crust. The asterisks denote models with lowered crustal geotherm.

**Extended Data Fig. 3:**
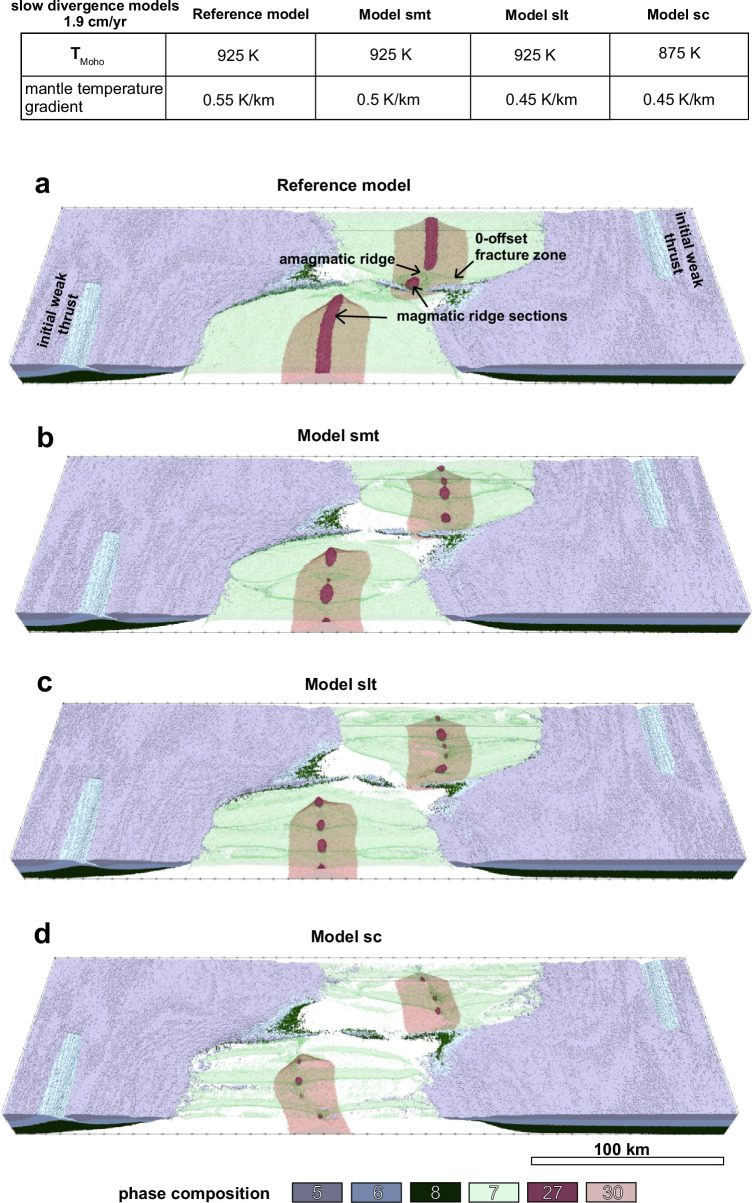
Additional numerical modelling results simulating 1.9 cm/yr total plate divergence. **a**, Reference model result. **b**, Model result with a lower, 0.5 K/km mantle thermal gradient. **c**, Model result with a lower, 0.45 K/km mantle thermal gradient. **d**, Model result with lower Moho temperature and a lower, 0.45 K/km mantle thermal gradient. Lower temperature values control suppressed melting, shorter magmatic ridge sections and larger volumes of the transform continental crust. Phase composition 5: continental upper crust, 6: continental middle crust, 7: oceanic upper crust (basalt), 8: continental lower crust, 27: molten basalt, 30: partially molten peridotite.

**Extended Data Fig. 4:**
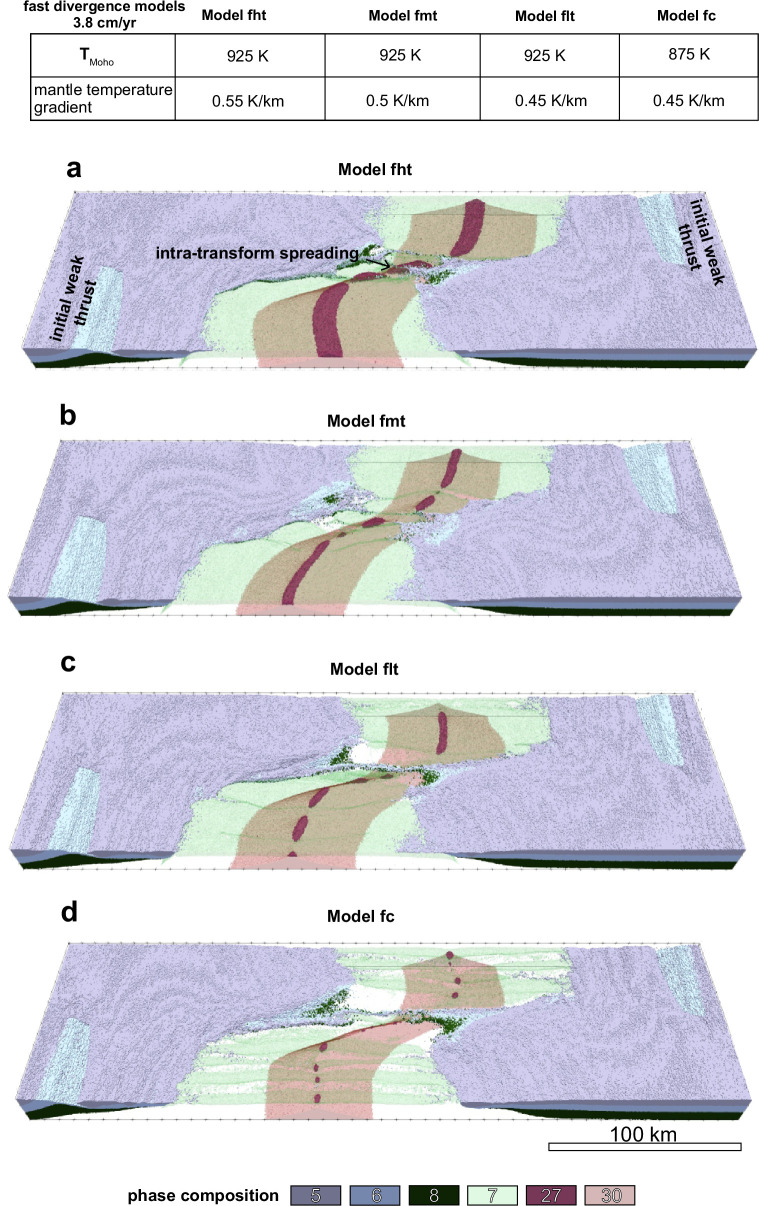
Additional numerical modelling results simulating 3.8 cm/yr total plate divergence. **a**, Model result with 0.55 K/km mantle thermal gradient. **b**, Model result with a lower, 0.5 K/km mantle thermal gradient. **c**, Model result with a lower, 0.45 K/km mantle thermal gradient. **d**, Model result with lower Moho temperature and a lower, 0.45 K/km mantle thermal gradient. Phase composition 5: continental upper crust, 6: continental middle crust, 7: oceanic upper crust (basalt), 8: continental lower crust, 27: molten basalt, 30: partially molten peridotite.

The plate divergence rate and mantle temperature gradients strongly influence the tectonic and magmatic evolution of transform zones and preservation of continental crustal slivers within them (Extended Data Figs. 3 and 4). Slower plate divergence (that is 1.9 cm yr^−1^) and lower temperature gradients are associated with suppressed mantle melting leading to mantle exhumation within the transform zone^48^. In these conditions, mid-ocean ridges containing magmatic and amagmatic segments slowly propagate towards the transfer zone, where the remaining continental bridge links the diverging rifted margins (Fig. 4a). The overlap geometry of the mid-ocean ridges governs a transient transpressional uplift of the crustal sliver, lasting approximately 2–4 Myr, before the fault zone reorganizes into a single transform fault, during drifting and thermal subsidence of the crustal slivers. Part of the former continental ridge remains attached to the rifted margins, forming similar geometries as the Newfoundland or East Greenland ridges, whereas narrower pieces of the continental crust and lithospheric mantle are transported and re-distributed along the oceanic transform and fracture zone. In contrast, faster plate divergence (that is 3.8 cm yr^−1^) and higher mantle thermal gradients enhance mantle melting resulting in fully magmatic mid-ocean ridges. In these cases, mantle melting also affects the transfer zone, forming magma chambers that eventually develop into an intra-transform oblique spreading centre, similar to the Siqueiros transform system^22^ (Fig. 4b). Here mantle melting and magma chamber formation occur before the propagating ridges reach the transfer zone, and the resulting oblique spreading centre disrupts the continental bridge. Over time, two oceanic transform faults bound the oblique spreading ridge, with only minor amounts of continental crust sheared within the transforms. Our models (Fig. 4 and Extended Data Figs. 3 and 4) infer that beyond the already explored intra-oceanic transform continental slivers, including the Romanche, Zabargad or the East Greenland cases, fragments of the continental crust and lithosphere may be preserved along other oceanic transform faults, depending on their rift history. Larger volumes of continental slivers are more likely to be preserved in regions with slower plate divergence and lower mantle temperature gradients (Fig. 5).

## Methods

### Bathymetric and gravity data

The presented maps of bathymetric and gravity data were compiled by GeoMapApp^49^ displaying GMRT 4.2 global bathymetry data and free air gravity anomaly maps^50^. The identification and interpretation of continental crustal slivers are based on available dredge haul and outcrop rock samples, such as in the Romanche and Zabargad examples, respectively. Furthermore, continental slivers are elevated from their surrounding oceanic basins driven by their lower densities and often uplifted by transpressional structures. Therefore, they show characteristic elevated and narrow, elongated bathymetric anomalies that are also reflected by high free air gravity anomalies. Finally, magnetic anomaly maps can be used to differentiate between the continental and oceanic crust, the latter showing magnetic anomalies aligned with the spreading ridges.

### Numerical modelling

The numerical thermomechanical 3D code I3ELVIS was used based on a combination of a finite-difference method, applied on a staggered Eulerian grid and a marker-in-cell technique^51,52^. The code solves the mass (equation (1)), momentum (equation (2)) and energy conservation (equation (3)) equations for visco-plastic incompressible media:

1 $$\frac{\partial {v}_{i}}{\partial {x}_{i}}=0$$

2 $$\frac{\partial {\sigma }_{{ij}}}{\partial {x}_{j}}-\,\frac{\partial {P}_{i}}{\partial {x}_{i}}=\,-\rho {g}_{i}$$

3 $$\rho {C}_{\mathrm{p},\mathrm{eff}}\frac{{\mathrm{D}T}}{{\mathrm{D}t}}=\,\frac{\partial }{\partial {x}_{i}}\left(k\frac{\partial T}{\partial {x}_{i}}\right)+\,{H}_\mathrm{r}+\,{H}_\mathrm{s}+\,{H}_\mathrm{a}+{H}_\mathrm{L}$$

4 $${H}_\mathrm{a}=T\alpha \,\frac{{\mathrm{D}P}}{{\mathrm{D}t}}$$

5 $${\rho }_{T,P}={\rho }_{0}(1-\alpha \left(T-{T}_{0}\right))(1+{\beta }_\mathrm{c}(P-{P}_{0}))$$

where $v$ is velocity, $\sigma$ is the deviatoric stress tensor, $P$ is the total pressure (mean normal stress), $\rho$ is the density, $g$ is the gravitational acceleration, ${C}_\mathrm{p,{eff}}$ is the effective isobaric heat capacity, $T$ is the temperature, $k$ is the thermal conductivity, which depends on pressure, temperature and rock composition, ${H}_\mathrm{r}$ is radioactive heating that is constant for a given rock composition, ${H}_\mathrm{s}$ is shear heating (product of deviatoric stress and strain rate), ${H}_\mathrm{a}$ is the adiabatic heating, ${H}_\mathrm{L}$ is latent heating, $\alpha$ is the thermal expansion and ${\beta }_\mathrm{c}$ is the coefficient of compressibility.

The code employs visco-plastic rheologies (Supplementary Table 1) including diffusion and dislocation creep. Plasticity is implemented using the following yield criterion ${\sigma }_{\mathrm{II}}\,\le \,{\sigma }_{\mathrm{yield}}$, which limits creep viscosity, altogether yielding an effective viscosity limit:

6 $${\eta }_{\mathrm{eff}}\le \frac{{\sigma }_{\mathrm{yield}}}{2{\dot{\varepsilon }}_{\mathrm{II}}}=\,\frac{{C}_{0}+{P}_\mathrm{e}\mathrm{sin}\varphi }{2{\dot{\varepsilon }}_{\mathrm{II}}}$$

where ${\eta }_{\mathrm{eff}}$ is the effective viscosity, ${C}_{0}$ is the cohesion, $\varphi$ is the friction angle, ${P}_\mathrm{e}$ is the effective pressure (total pressure subtracting the hydrostatic fluid pressure) and ${\dot{\varepsilon }}_{\mathrm{II}}$ is the second invariant of strain rate. Plastic strain weakening^53^ is implemented by linearly decreasing the cohesion and friction angle over the strain interval of 0.1–1. A constant plastic healing rate (10^−14^ s^−1^) is applied to heal deactivated shear zones over time^51^.

The model accounts for simplified magma-related processes, such as thermal accretion of the lithospheric mantle, partial melting of the mantle assuming a simplified parameterized model^54^, melt extraction and percolation towards the ridge, crystallization of the oceanic crust. Lagrangian markers track the amount of melt extracted during model evolution. Furthermore, hydrothermal circulation at the axis of the ridge is parametrized with an enhanced thermal conductivity of the oceanic crust resulting in its rapid cooling. Further description of these processes and their sensitivity analysis is written in previous studies^7,20,51,52,55^.

Erosion and sedimentation are simulated by the coupled finite differences surface processes model^56^ to the thermo-mechanical code (FDSPM-I3ELVIS). Model results are visualized by Paraview. Our models focused on the first-order processes controlling continental rifting and oceanic spreading and limitations are discussed here. Our 3D set-up simulates only a limited model domain of 484 × 196 × 228 km^3^ with 1-km resolution and a laterally uniform initial crustal and lithospheric configuration was designed. We applied constant velocity boundary conditions; variable velocities are tested in previous models^52^. A larger model domain would enable us to study in details the rift segments evolution before rift linkage and the formation of microcontinents. Our application of erosion and sedimentation by diffusion is an advancement in comparison to previous similar 3D models without surface processes. However, the simulation of more realistic basin-scale stratigraphy would require further approaches^57^. Our simulations include mantle melting and oceanic crust formation by magmatic (plutonic) accretion from the axial magma region^55^, but dykes emplacement into the crust and volcanic crustal growth that would alter the heat flow evolution are not considered here. Finally, our models did not include elastic rheology. Elastic flexure alters rift morphology, fault life span and subsidence history^58^, which can be particularly important when addressing erosion and sediment re-deposition processes. Our newest model results on testing visco-elasto-plastic rheology^59^, however, show a similar evolution of continental slivers emplacement into oceanic transform fault zones.

## Online content

Any methods, additional references, Nature Portfolio reporting summaries, source data, extended data, supplementary information, acknowledgements, peer review information; details of author contributions and competing interests; and statements of data and code availability are available at 10.1038/s41561-025-01795-0.

## Supplementary information

Supplementary Information Supplementary Figs. 1–4, Table 1 and caption for Supplementary Videos 1–4.

Supplementary Video 1 Crustal evolution of the reference model.

Supplementary Video 2 Effective viscosity evolution of the reference model.

Supplementary Video 3 Crustal evolution of the model with faster divergence velocity and higher mantle temperature gradient (model fht).

Supplementary Video 4 Effective viscosity evolution of the model fht.

## Data Availability

All the relevant data and model outputs presented in this study are available via Zenodo at 10.5281/zenodo.16793916 (ref. ^60^) and in the Supplementary Information.

## Extended data

**Extended Data Table 1:** List of intra-oceanic continental slivers

Continental sliver	Transform stage	Sliver style	Geometry	Supporting data	References
Romanche continental slivers	Mature, large-offset oceanic megatransform	Two blocks detached from the rifted continental margins, bounded by oceanic transform faults	2-12 km wide, elongated slivers with prominent bathymetric spurs	Seismic profiles; pre-rift continental sediments and metasediments dredged from the elevated transform ridge	^12,13,27,61,62^
Zabargad continental island	High-offset Zabargad fracture and proto-transform segments bounding the northern and central Red Sea	Elevated continental block within a segmented transform fault zone	~ 5 km wide island	Outcropping Pan-African granitic gneiss on the island	^14,28,29^
East Greenland Ridge	Fossil continental transform margin predating the evolution of the Knipovich Ridge	Crustal sliver attached to the rifted margin of Greenland	~ 200-250 km long, 30 km wide, continental ridge	Detailed seismic imaging and gravity modelling	^15,63^
Newfoundland Ridge	Continental ridge formed during the evolution of the southern Newfoundland transform margin	Extended continental ridge attached to the North American rifted margin	Broad bathymetric spur along the southern Newfoundland transform margin	Detailed seismic imaging	^31^
Davie crustal sliver	Transform fault zone associated with the southward drift of Madagascar from eastern Africa. Interpreted as a continental bridge remnant.	Shallow-bathymetric transpressional ridge along the transform fault zone of the Davie Fracture Zone.	~ 0-50 km wide ridge	Reflection seismic interpretation and gravity modeling; pre-rift continental basement rocks dredged from elevated segments of the ridge	^32,33,64,65^
Woodlark Basin sliver	Active continental transform margin and oceanic transform fault zone formation	Continental spurs along the transform margins, likely still attached to the rifted margin.	Narrow, V-shaped thinned continental spurs	High-resolution bathymetry and potential geophysical interpretations	^34^

## Code availability

The numerical code I3ELVIS is available via Zenodo at 10.5281/zenodo.16793916 (ref. ^60^). 3D data visualization was carried out by Paraview.
