# Supplementary material for: Presence of continental slivers in oceanic transform faults determined by rift inheritance
Source: Nat Geosci. 2025 Sep 25;18(12):1303–10. doi: 10.1038/s41561-025-01795-0 (PMC12685745; doi:10.1038/s41561-025-01795-0)
Supplement: Supplementary file 1 — Supplementary Figs. 1–4, Table 1 and caption for Supplementary Videos 1–4. [file 41561_2025_1795_MOESM1_ESM.pdf]

nature geoscience

Supplementary information https://doi.org/10.1038/s41561-025-01795-0

Presence of continental slivers in oceanic transform faults determined by rift inheritance

In the format provided by the authors and unedited

Contents:

Supplementary figures

Supplementary table

Caption for supplementary videos

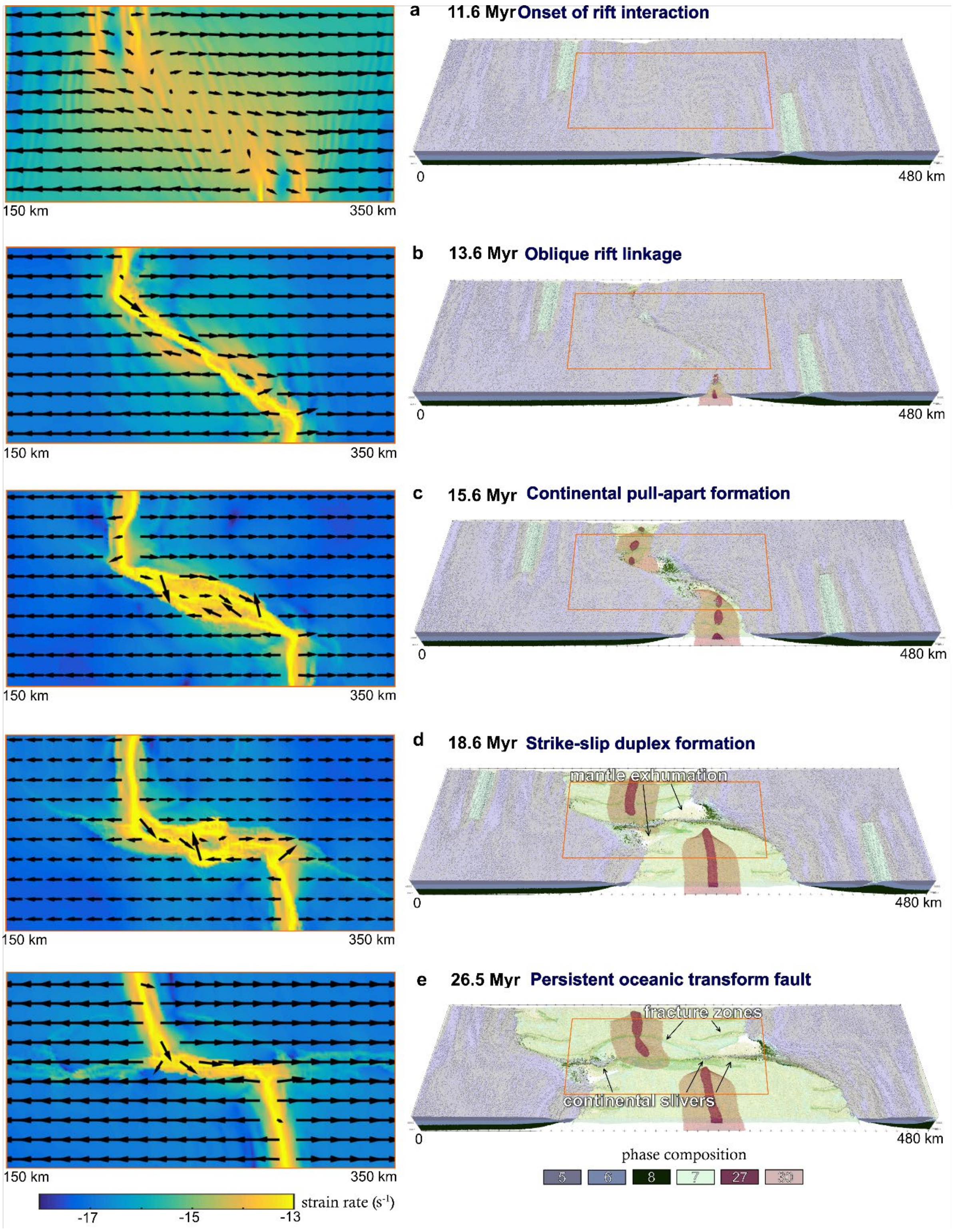

Fig. S1 Reference model evolution of the formation and evolution of continental slivers in oceanic transform faults. Results are simulated by 3D magmatic-thermo-mechanical geodynamic and coupled surface processes models and shown by strain rate and velocity field on horizontal maps and rock composition (setup is shown in Extended Data Fig. 2). Sediments are transparent in this figure. WZ: inherited weak fault zones in the crust. Phase composition 5: continental upper crust, 6: continental middle crust, 7: oceanic upper crust (basalt), 8: continental lower crust, 27: molten basalt, 30: partially molten peridotite.

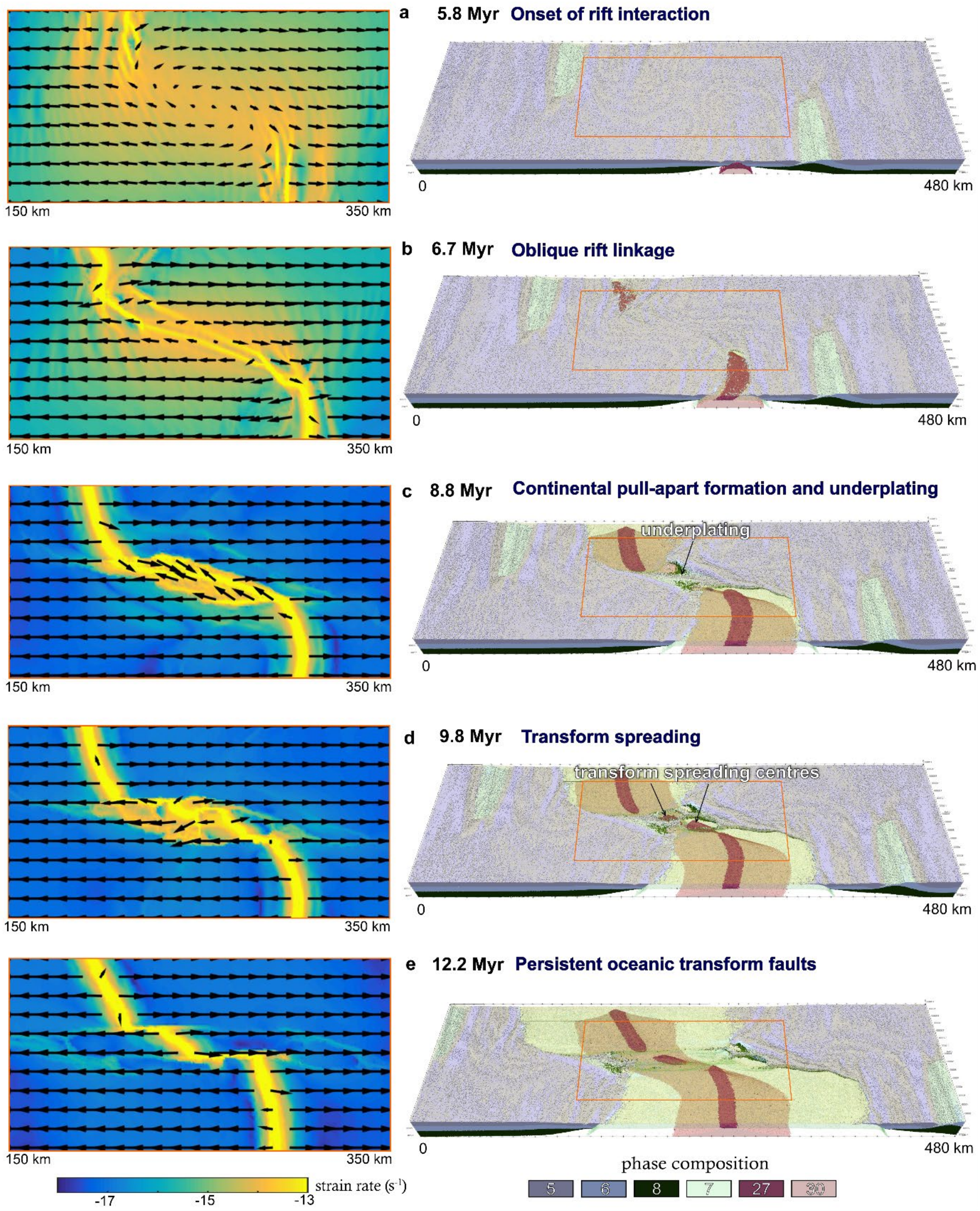

Fig. S2 Evolution of an intra-transform spreading ridge. Results of model fht are simulated by 3D magmatic-thermo-mechanical geodynamic and coupled surface processes models and shown by strain rate and velocity field on horizontal maps and rock composition (setup is shown in Extended Data Fig. 2). Sediments are transparent in this figure. WZ: inherited weak fault zones in the crust. Phase composition 5: continental upper crust, 6: continental middle crust, 7: oceanic upper crust (basalt), 8: continental lower crust, 27: molten basalt, 30: partially molten peridotite.

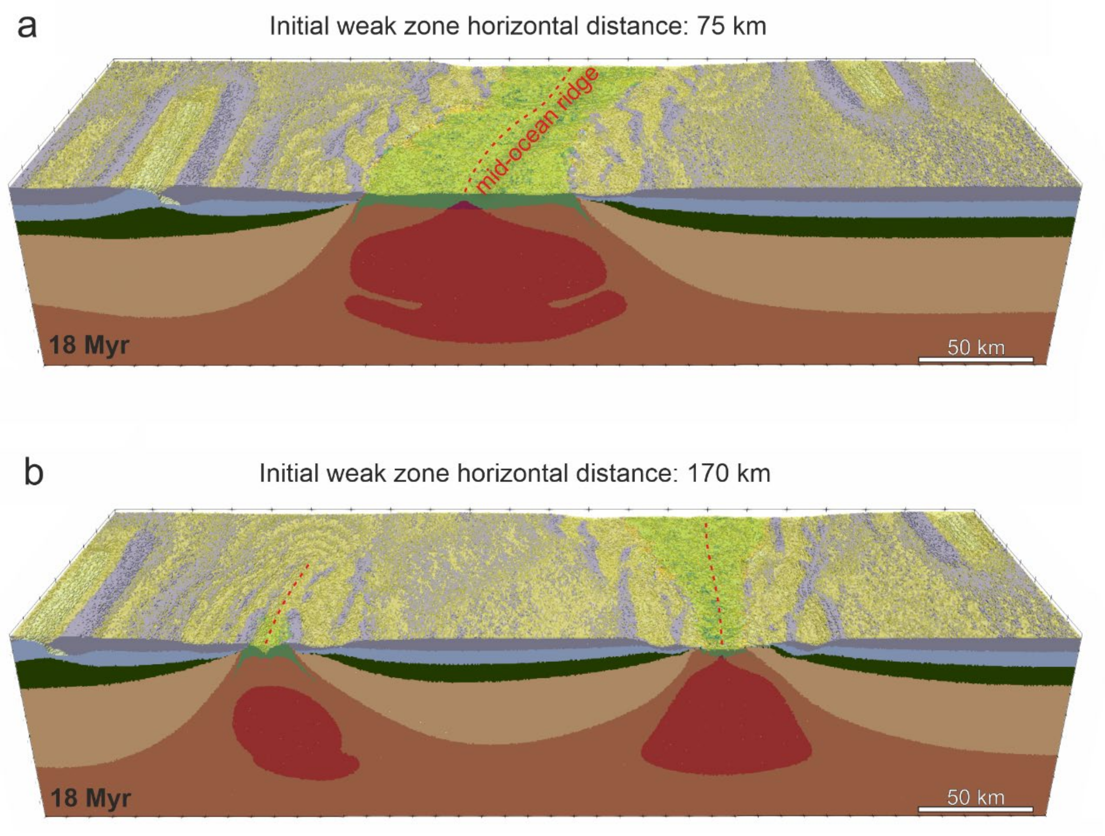

Fig. S3 Model results showing end-member scenarios of rift linkage. Results are simulated by 3D magmatic-thermo-mechanical geodynamic and coupled surface processes models and shown by rock composition (setup is shown in Extended Data Fig. 2). **a,** Development of a continuous, oblique mid-ocean ridge. **b**, Development of two disconnected mid-ocean ridges and oceanic basins bounding a continental domain in the centre. The efficiency of rift and ridge linkage and interaction is driven by the efficiency of the stress transfer and strain localization in the crust and lithosphere linked to lithosphere rheology, geometrical parameters, such as the horizontal distance of the ridges and also influenced by the applied boundary conditions and model dimensions.

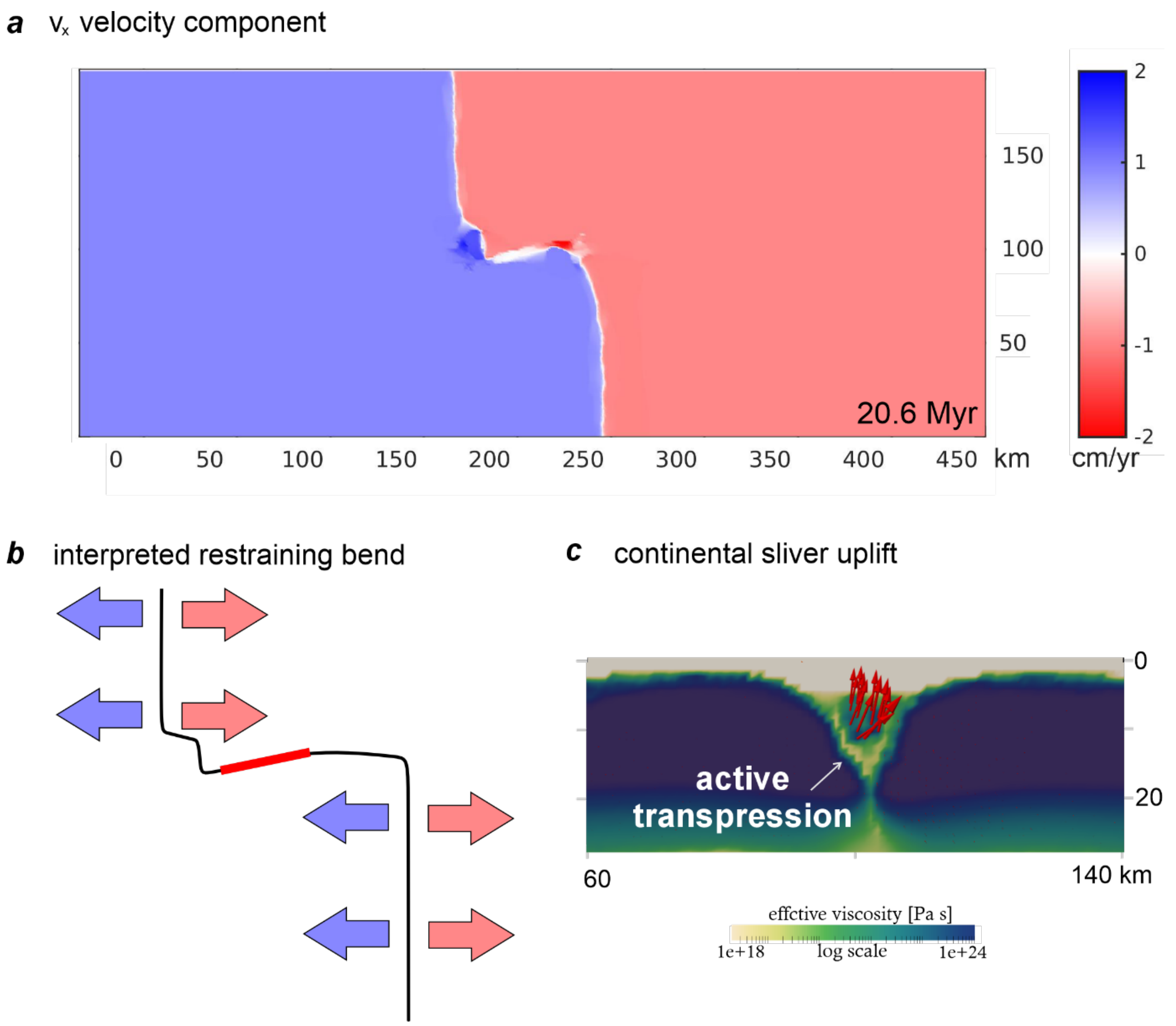

Fig. S4 Model results highlighting the transpressional stage of oceanic transform fault zone evolution. a, Upper crustal velocity map showing vx velocity component after 20.6 Myr of the reference model. **b,** Sketch showing the development of a restraining bend between the mid-ocean ridge segments. **c,** Effective viscosity vertical cross section through the middle of the model domain overlain by velocity vectors in red showing the uplift of the continental sliver.

	Upper cont. crust	Middle cont. crust	Lower cont. crust	Lithospheric mantle	Sediments	Mantle Weak zone	Basalt
Rheology	wet quartzite	wet quartzite	plagioclase	dry olivine	wet quartzite	wet olivine	plagioclase
Density, ρ_0 (kg m^{-3})	2750	2750	2800	3300	2600	3300	3000
Pre-exponential factor, $1/A_D$ (Pa^n s)	1.97×10^{17}	1.97×10^{17}	4.8×10^{22}	3.98×10^{16}	1.97×10^{17}	3.98×10^{16}	4.8×10^{22}
Activation energy, E (kJ mol^{-1})	154	154	238	532	154	470	238
Power law exponent, n	2.3	2.3	3.2	3.5	2.3	4.0	3.2
Cohesion (Pa)	$10 - 3 \times 10^6$	$10 - 3 \times 10^6$	$10 - 3 \times 10^6$	$10 - 3 \times 10^6$	1×10^6	$3 - 1 \times 10^6$	$10 - 3 \times 10^6$
Coefficient of friction, sin(ϕ)	0.6-0.2	0.6-0.2	0.6-0.2	0.6-0.0	0.2-0.0	0.1-0	0.6-0
Radioactive heat production, H_r (μW/m^3)	2	2	0.2	0.022	0.024	0.022	0.022

Table S1. Main rheological parameters of the experiments after Ranalli (1995), Gerya (2015) and Balazs et al. (2023).

Gerya T.V., Stern, R.J., Baes, M., Sobolev, S.V. & Whattam S.A. Plate tectonics on the Earth triggered by plume-induced subduction initiation. Nature 527, 221-225 (2015).

Ranalli, G. Rheology of the Earth, 2nd edn. Chapman and Hall, (1995).

Balazs, A., Gerya T.V., May, D. & Tari, G. Contrasting transform and passive margin subsidence history and heat flow evolution: insights from 3D thermo-mechanical modelling. In: Geol. Soc. Lond. Spec. Pub. 524, 191-217. Tectonic Development, Thermal History and Hydrocarbon Habitat Models of Transform Margins: their Differences from Rifted Margins, eds. Nemčok, M. et al. (2023).

Video S1. Crustal evolution of the reference model.

VideoS2. Effective viscosity evolution of the reference model.

VideoS3. Crustal evolution of the model with faster divergence velocity and higher mantle temperature gradient (model fht).

VideoS4. Effective viscosity evolution of the model fht.
