# Supplementary figures and images for: Presence of continental slivers in oceanic transform faults determined by rift inheritance
Source: Nat Geosci. 2025 Sep 25;18(12):1303–10. doi: 10.1038/s41561-025-01795-0 (PMC12685745; doi:10.1038/s41561-025-01795-0)

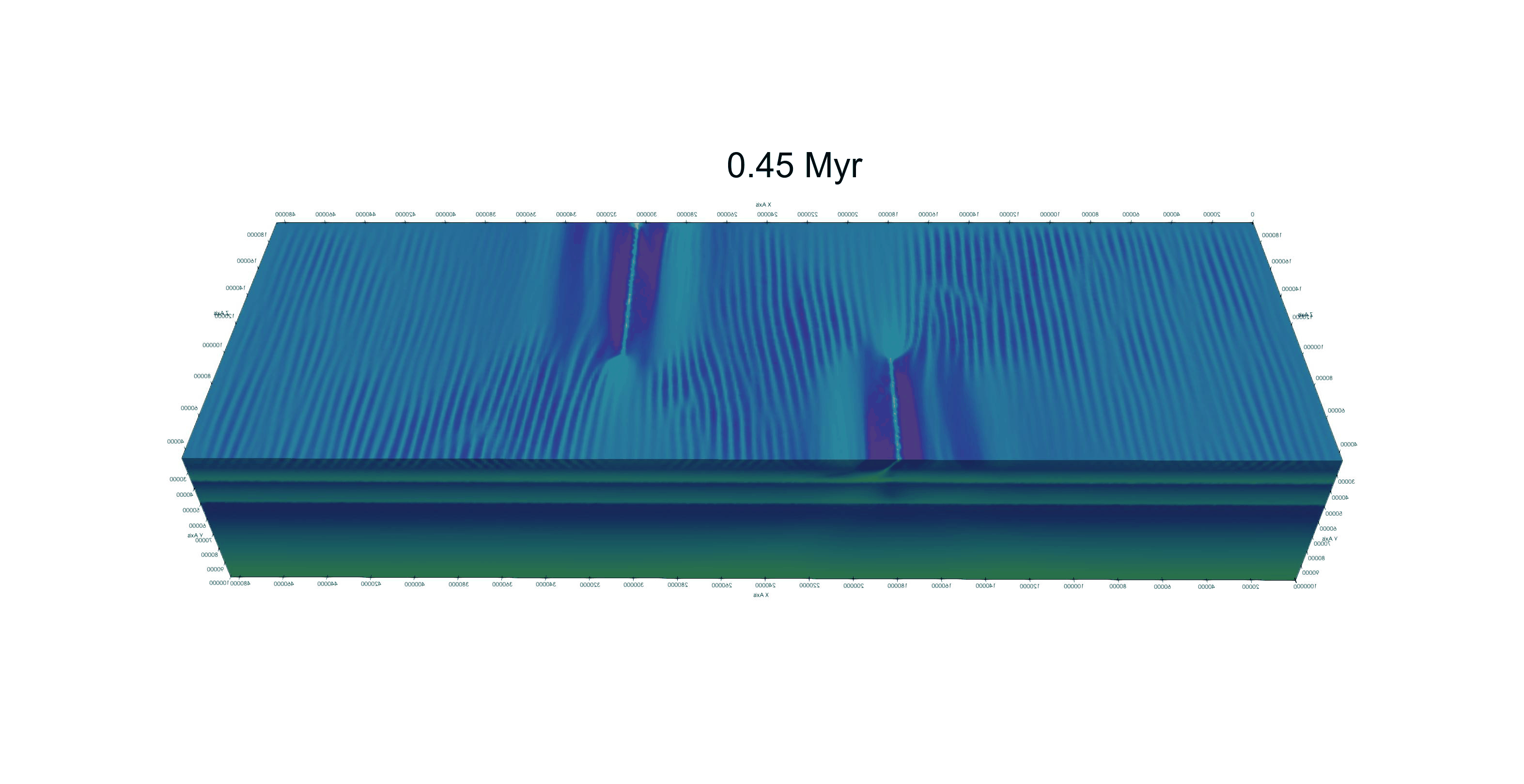

Supplement: Supplementary file 3 — Effective viscosity evolution of the reference model. [file 41561_2025_1795_MOESM3_ESM.gif]

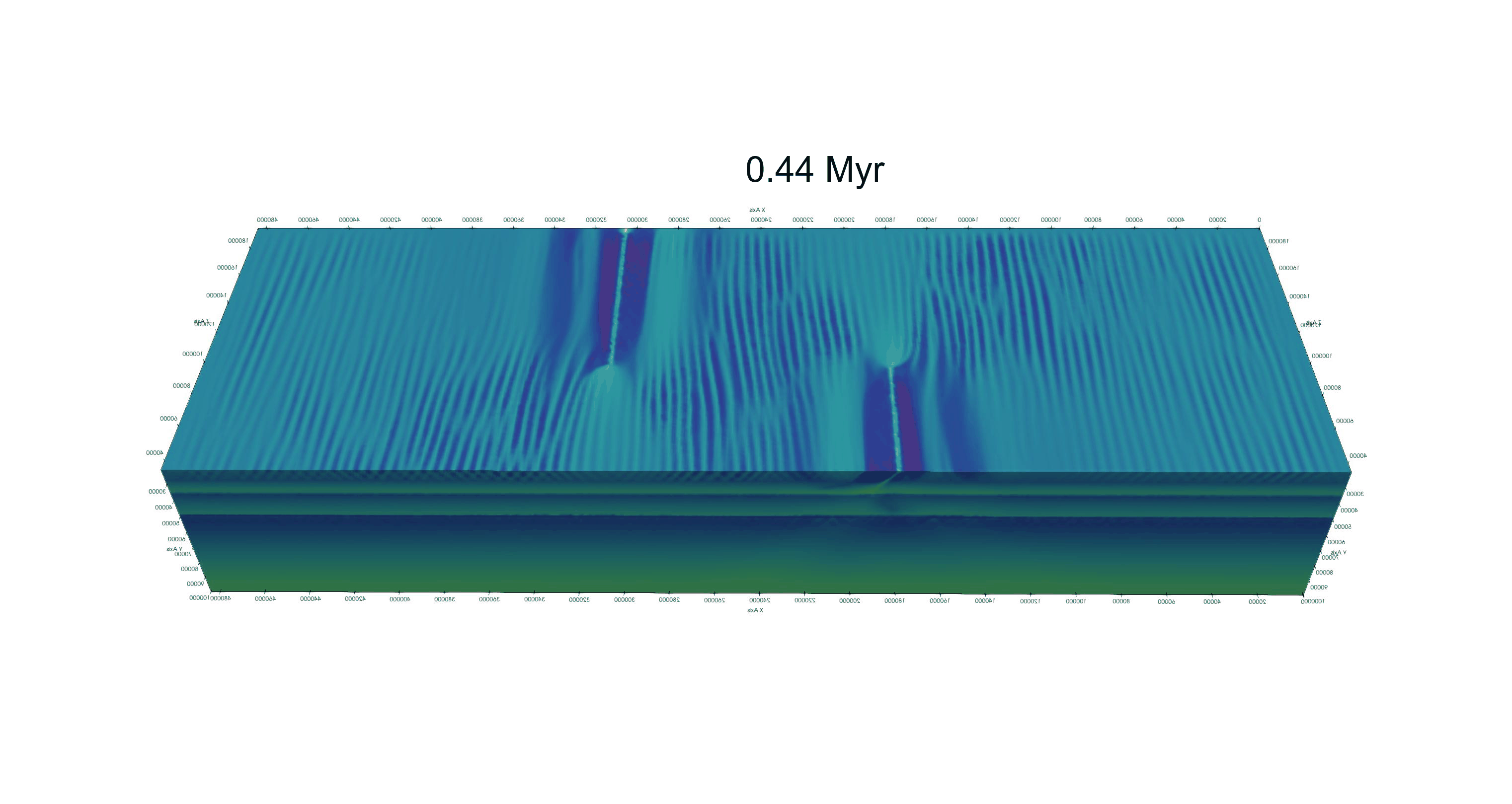

Supplement: Supplementary file 5 — Effective viscosity evolution of the model fht. [file 41561_2025_1795_MOESM5_ESM.gif]
